# Reducing Inappropriate Urinary Catheter Use by Involving Patients Through the Participatient App: Before-and-After Study

**DOI:** 10.2196/28983

**Published:** 2022-04-04

**Authors:** Robbert G Bentvelsen, Marguerite L Bruijning, Niels H Chavannes, Karin Ellen Veldkamp

**Affiliations:** 1 Department of Medical Microbiology Leiden University Medical Center Leiden University Leiden Netherlands; 2 Microvida Laboratory for Medical Microbiology Amphia Hospital Breda Netherlands; 3 Department of Public Health and Primary Care Leiden University Medical Center Leiden University Leiden Netherlands

**Keywords:** infection control, catheter-associated urinary tract infections, urinary catheter, patient empowerment, catheter, urology, infection, urinary tract infection, smartphone app, surgical nursing

## Abstract

**Background:**

The risk of urinary tract infections is increased by the inappropriate placement and unnecessary prolongation of the use of indwelling urinary catheters. Sustained behavior change in infection prevention could be promoted by empowering patients through a smartphone app.

**Objective:**

The aim of this study is to assess the feasibility and efficacy of implementation actions on patients’ use of the Participatient app on a clinical ward and to compare 3 survey methods for urinary catheter use.

**Methods:**

Participatient was introduced for all admitted patients at the surgical nursing ward in a university hospital in the Netherlands. Over a period of 3 months, the number of new app users, days of use, and sessions were recorded. In a comparison of urinary catheter use before and after the implementation of the app, 3 methods for point prevalence surveys of catheter use were tested. Surveys were conducted through manual parsing of the text in patients’ electronic medical records, parsing a survey of checkbox items, and parsing nursing notes.

**Results:**

In all, 475 patients were admitted to the ward, 42 (8.8%) installed the app, with 1 to 5 new users per week. The actions with the most ensuing app use were the kick-off with the clinical lesson and recruiting of the intake nurse. Between the survey methods, there was considerable variation in catheter use prevalence. Therefore, we used the standard method of manual parsing in further analyses. Catheter use prevalence decreased from 38% (36/96) to 27% (23/86) after app introduction (OR 0.61, 95% CI 0.32-1.14).

**Conclusions:**

The clinical application of Participatient, the infection prevention app for patients, could be feasible when implementation actions are also used. For surveying indwelling urinary catheter use prevalence, manual parsing is the best approach.

## Introduction

The risk of urinary tract infections increases with the inappropriate placement and unnecessary prolonged use of indwelling urinary catheters. As a result, catheter-associated urinary tract infections (CAUTIs) are a leading cause of health care–associated infections (HAIs). In Europe, CAUTIs account for 152 (95% CI 145-161) cases of HAIs and 81.2 (95% CI 69.0-94.2) disability-adjusted life years per 100,000 population per year. CAUTIs cause excess morbidity, increased length of hospital stays, and increased use of antibiotics [1,2].

Current infection control programs for CAUTI prevention increase health care worker knowledge and awareness of inappropriate catheter use with varying success. Best practices for preventing CAUTIs in acute care hospitals include providing guidelines, supplies, and documentation; performing CAUTI surveillance; educating and training staff; ensuring the use of appropriate technique for aseptic insertion; and ensuring appropriate management [3,4]. Sustained behavior change in appropriate catheter use is often arduous [5]. However, although challenging to implement and compare, we hypothesize that patient participation in infection prevention could be an effective sustainable strategy [6].

Together with patients, nurses, and physicians, we initially developed the smartphone app Participatient for patients. It contains details on practical matters related to the hospital stay, such as visiting hours, and a catheter check function aiming to promote communication on catheter use [7]. The catheter check helps patients assess the indication for their catheter. If no appropriate indication is found, the app advises the user to ask their nurse or physician for the indication. This way, the app helps create awareness and reduce the unnecessary (long-term) use of catheters, thereby aiming to reduce CAUTIs on the entire ward. Surveys of catheter use will be essential for testing the eHealth intervention Participatient in the future. Therefore, in addition to the standard manual survey, 2 alternative methods for surveying catheters will also be evaluated. If accurate enough compared to the standard method, parsing of checkboxes or nurses’ notes parsing methods could be a more efficient alternative to manual text parsing.

This study is embedded in the National eHealth Living Lab (NeLL). Through interdisciplinary collaboration, eHealth studies conducted in partnership with the NeLL will create new and innovative solutions that improve health and wellbeing by using suitable eHealth tools for each specific research question.

In this study, the main objective was to assess the feasibility of implementing the Participatient app and the efficacy of sequential stimulating actions on patients’ use of the app. The secondary objective was to compare the accuracy of 3 survey methods for urinary catheter use.

## Methods

### Study Design and Setting

In this study, a before-and-after design was used; surveys were conducted on indwelling urinary catheter use before (T0) and after (T1) the app was introduced. Usage data were continuously collected from the app after introduction.

Adult patients were eligible for inclusion in the surveys if admitted to the 36-bed nursing ward for general, gastrointestinal, and oncological surgery at the Leiden University Medical Center in the Netherlands. Patients were excluded from the point prevalence surveys (PPS) if they were not present on the ward at the time of the survey or if they were admitted on the day of the survey. Patients in T1 were invited to use the app during their stay.

### Ethics Approval

This trial was approved by the Medical Ethics Research Committee of the Leiden University Medical Center, with a waiver for individual informed consent (protocol C17.075). Local feasibility was approved by the ward.

### Participatient App

**Figure 1:**
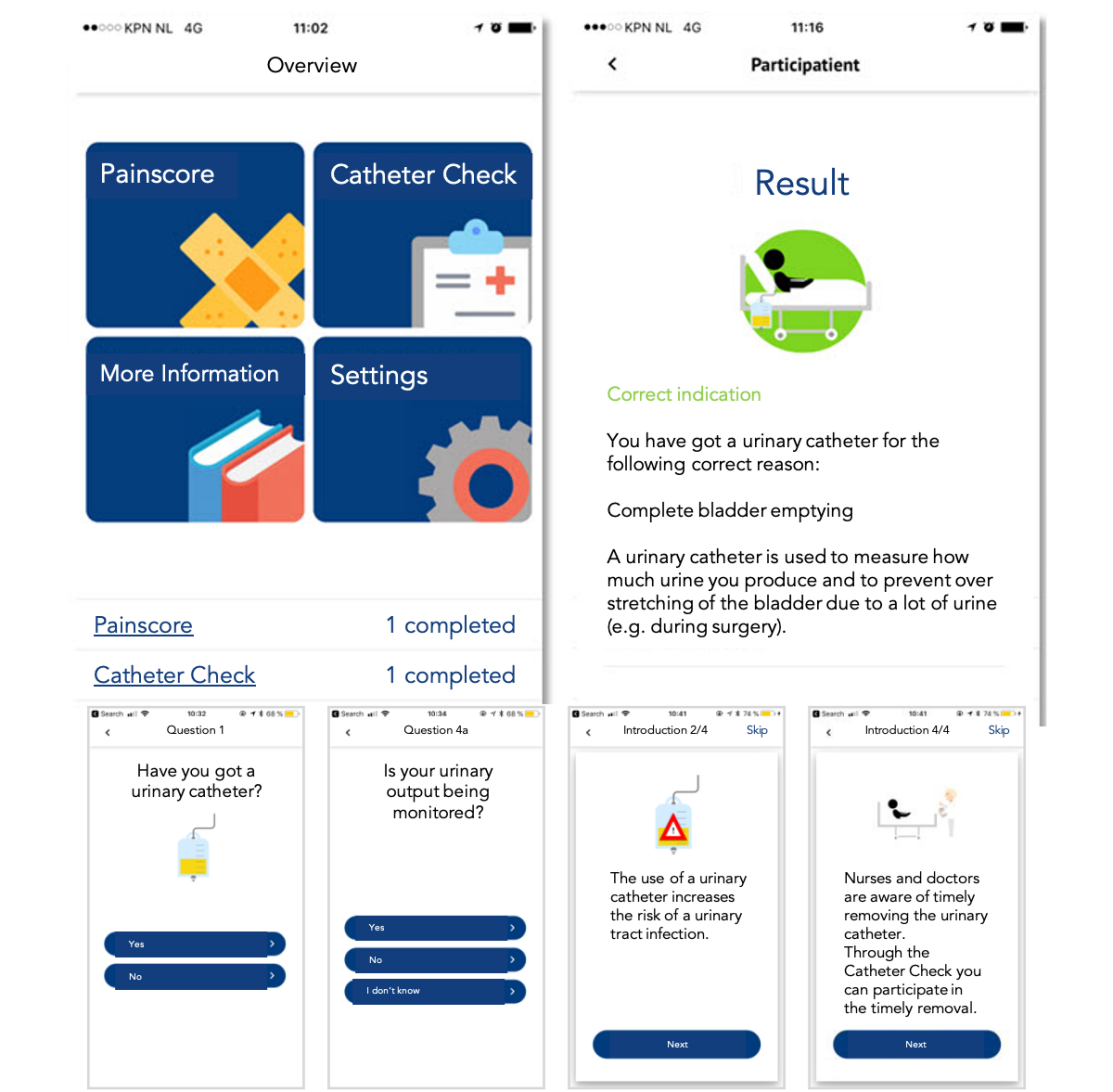
The Participatient app with the catheter check function.

The top left panel of Figure 1 shows the Participatient app menu with links to the following pages: painscore (added to help with pain management during admission), catheter check, more information, and settings. The top right panel of Figure 1 shows the result screen for the catheter check function with, depending on the outcome of the questionnaire, an appropriate indication for catheter use (measuring urinary output) with background information. The bottom row of Figure 1 shows screenshots of the questions in the catheter check function with an explanation and a prompt for the patient to discuss their catheter use with their nurse or physician. App development and the final product were previously described in full [7].

### Feasibility Testing of the Implementation Actions

In preparation for putting this previously developed app (Figure 1) into practice, the nursing team was given a clinical lesson on urinary tract infections, catheter indications, patient involvement, and the functions of the Participatient app (this implementation action is hereby referred to as the kick-off with the clinical lesson). The nurses were asked to provide input for updating the app and for adjusting it to the ward. Adjustments included the addition of visiting hours, staff, and medical information. After updating the app with these adjustments, nurses merely invited patients to download the app upon admission to the ward. As an implementation action during the course of the study, the intake nurse was recruited and trained to invite patients early in the patients’ admission to the ward to further stimulate app use. Additionally, the researchers scheduled reminders of the project for the nurses to promote app use (stimulant reminder given to nurses).

The efficacy of the implementation actions on app use was evaluated by calculating the number of new users, sessions, and days of active use. A new user was registered when the catheter check was used on a unique mobile device. Every instance of opening the app was counted as a session. The days of active use were calculated as the days with one or more sessions per user. Access to the app was restricted to the ward through a 4-digit code.

### Feasibility Testing of the 3 Survey Methods

The PPS were conducted using 3 methods and the accuracy of these methods was compared. Data were collected on the prevalence, indication, and duration of urinary catheter use. The PPS were carried out according to national and international guidelines [8,9], as was the scoring of the appropriateness of the catheter indication (Multimedia Appendix 1).

The prevalence and indication of catheter use were manually scored as documented in-text on the date of the survey in the electronic medical record (EMR). This method was compared with the sensitivity and specificity of surveys of checkboxes (which could be automated) and with parsing nursing notes surveys (Table 1 and Multimedia Appendices 2-4). In the event of missing data on the catheter indication, the reason for catheter use was scored as “not registered” and thus, inappropriate. A total of 2 trained observers (MLB and RGB) independently surveyed catheter use to reduce bias in the measurement of the outcome [10]. We compared the results and discussed discrepancies with a senior observer (KEV).

Data were analyzed using descriptive statistics with the statistical package SPSS (version 26.0; IBM Corp). Between categorical variables, associations were tested with the Pearson chi-squared test, calculating odds ratios with confidence intervals and *P* values. Two-sided *P* values less than .05 were considered significant.

**Table 1:** A comparison of the 3 survey methods for urinary catheter use before (T0) and after (T1) the implementation of the Participatient app.

Survey methods	Urinary catheter prevalence at T0 (n=96), n (%)	Urinary catheter prevalence at T1 (n=86), n (%)	Odds ratio (95% CI)	*P* value^a^
Manual text parsing	36 (38)	23 (27)	0.61 (0.32-1.14)	.12
Checkbox survey parsing	37 (39)	29 (34)	0.81 (0.44-1.49)	.50
Nurses’ notes parsing	28 (29)	18 (21)	0.64 (0.33-1.27)	.20

^a^Two-sided *P* values less than .05 were considered significant.

## Results

### App Use

The Participatient app was introduced between October 2017 and January 2018 (T1). Of the 475 patients admitted to the ward, 42 (8.8%) new users installed and used the catheter check function. We registered 85 days of active use and 156 sessions, with an average use of 3.7 sessions per individual user (Multimedia Appendix 5). Since the app is meant to be privacy-friendly, we did not collect demographic data on users.

**Figure 2:**
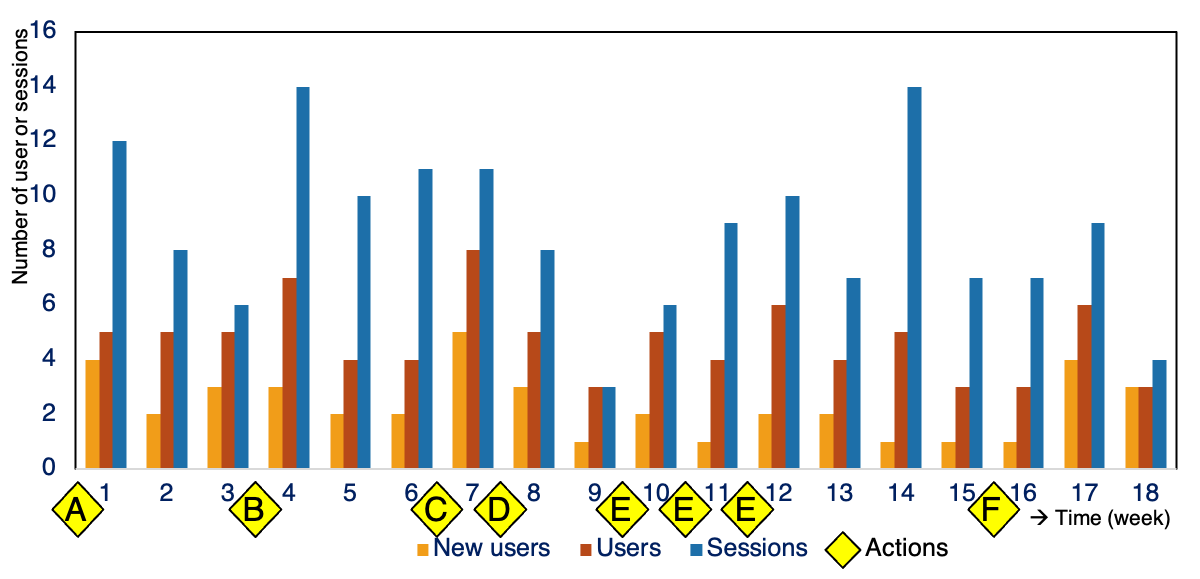
Results of implementation actions to increase patients’ app use. The efficacy of the implementation actions was registered as the number of new users, users, and sessions.

The highest number of new users was seen following the kick-off with the clinical lesson for nurses and after recruiting the intake nurse to promote app use for the project. The infographic posters and the stimulant reminder given to nurses resulted in the fewest new users among the implementation actions used (Figure 2). The app users reported to be very satisfied with their involvement and the personalized advice they received, rating the app 4.7 out of 5 stars.

The efficacy of the implementation actions for promoting app use was registered as the number of new users, users, and sessions.

A new user was measured as a new installation on a unique mobile device. The number of users referred to the total number of unique users per 24 hours, and a session was counted for every instance of use. Within Figure 2, the following implementation actions are marked at the time point when they began: (A) kick-off with the clinical lesson, (B) infographic posters, (C) recruiting the intake nurse, (D) stimulant reminder given to the nursing team, (E) support rounds with technical assistance performed by the research team, and (F) feedback given to the nursing team on app use and catheter use prevalence. Actions C and D were added to the scheduled actions after interim analyses and feedback from the ward.

### Catheter Use Surveys

Of the 182 patients included in the PPS, 96 patient records were surveyed in T0 and 86 were surveyed in T1. Baseline characteristics (sex and age) were similar between the groups (Multimedia Appendix 6). The mean age was 63 years, and 41% (75/182) of patients were female.

The prevalence of indwelling urinary catheter use differed considerably between survey methods (Table 1 and Multimedia Appendices 2-4). Compared to the customary method of manual parsing of the text in the EMR, parsing a survey of checkbox items in the EMR had a sensitivity of 96.6% and a specificity of 92.7%. Paring nurses’ had a sensitivity of 64.4% and a specificity of 93.5%. Therefore, we decided to continue to use manual parsing in further analyses.

Catheter use prevalence on the ward decreased from 38% (36/96) of patients to 27% (23/86) of patients after app introduction (OR 0.61, 95% CI 0.32-1.14). The average duration of catheterization dropped from 6.9 days to 2.3 days, while the median remained 2 days. We found a 39% (from 56% to 17%) decrease in the number of inappropriate indications for catheter use after the introduction of the app (OR 0.17, 95% CI 0.05-0.60). A total of 56% (20/36) of patients had an inappropriate indication before the introduction of the app, and 17% (4/23) had an inappropriate indication after the introduction of the app. This is shown in Table 2.

**Table 2:** The number of incorrect indications for catheter use before (T0) and after (T1) the implementation of the Participatient app.

Time point of inappropriate indication	Urinary catheter prevalence at T0 (n=36), n (%)	Urinary catheter prevalence at T1 (n=23), n (%)	Odds ratio (95% CI)	*P* value^a^
At catheter insertion	10 (28)	0 (0)	N/A^b^	.006
At catheter survey	20 (56)	4 (17)	0.17 (0.05-0.60)	.004

^a^Two-sided *P* values less than .05 were considered significant.

^b^Not applicable due to an inappropriate urinary catheter prevalence of 0% at T1.

## Discussion

The Participatient app aims to reduce the inappropriate (long-term) use of indwelling urinary catheters through patient involvement. The primary objective of this study, to assess the feasibility of the implementation and efficacy of implementation stimulating actions on the use of the Participatient app, was achieved by registering new users each week. Additionally, we were able to compare the 3 survey methods for catheter use.

A total of 1 in 11 patients admitted to the ward used the app. The highest number of new app users per week was registered following the kick-off with the clinical lesson and after recruiting the ward’s intake nurse. The peak in downloads in weeks 17 and 18 could be a delayed result of the feedback given to nurses in week 16. We hypothesize that actively engaging the nurses increases their motivation to promote the app. This is largely consistent with patient involvement in infection prevention, which increases with explicit permission to use the app and participate in their care by staff. As in the other HAI prevention studies, we found that involving nursing staff and keeping them engaged through multifaceted stimulant actions is essential for patient empowerment [4,6,11,12].

Parsing the survey of checkbox items or parsing nursing notes for indwelling urinary catheter use prevalence was inadequate compared to manual text parsing. Manual EMR parsing is laborious; however, this method is most in line with guidelines and previous studies [4,8,9] and is needed for the assessment of the catheter indication. Additionally, the results of the alternative survey methods (parsing checkboxes or nursing notes) are too far removed from the standard method. This could be due to a failure to register the catheter removal date in the proper EMR entry field or updating the daily nursing note too late.

A strength of this study is that it assesses the innovative approach of using eHealth to reduce CAUTIs. Additionally, engaging with physicians, nurses, and patients provides a relevant new perspective. A possible limitation of this study is the manual survey method with the registration of indications for catheter use is limited to parsing of the text in the EMR. Indications not described could have been missed. Additionally, the results could be biased by the introduction of the app to a specific department and/or the small sample size. Registered use of the catheter check function was only 8.8% (42/475), with the target group being elderly hospitalized patients. This was not unexpected as the app should be seen as part of a bundle of interventions to create awareness on the ward as a whole. Encouraging patients to use the app and employing nurse ambassadors who can promote the app could help improve app use. Furthermore, CAUTIs, which are also a relevant outcome, were not scored as this was not the objective due to the short duration of this feasibility study. Remarkably, the prevalence of urinary catheter use was high, with a decreasing trend after the introduction of the app. Additionally, the fraction of inappropriately used catheters decreased significantly.

Jones et al [13] found interventions aimed at the prevention of CAUTIs and *Escherichia coli* bacteremia often did not use behavioral theory or frameworks, and research is required using robust methodologies to evaluate these interventions. The Participatient intervention is designed and built according to the CeHRES (Centre for eHealth and Wellbeing Research) framework, with the involvement of all stakeholders in the development [7]. The before-and-after study design used for this feasibility study could be improved to decisively conclude on the intervention’s effectiveness. In mHealth interventions, economic evaluations are limited. In future assessments, this should be included in the analysis [14].

The clinical application of Participatient, the infection prevention app for patients, could be feasible when implementation actions are combined. Engaging physicians and nurses could help because additional users are observed after the implementation stimulating actions, particularly when actively involving nursing staff. Manual parsing is the preferred method for surveying the effect on urinary catheter use. A larger study spanning various populations could further evaluate the app’s effectiveness with the outcomes of catheter use appropriateness and infections.

## Appendix

Multimedia Appendix 1 Urinary catheter indications.

Multimedia Appendix 2 A detailed comparison of 3 survey methods for urinary catheter use before (T0) and after (T1) implementation of the Participatient app.

Multimedia Appendix 3 A comparison of the following survey methods for urinary catheter use: manual parsing versus checkboxes.

Multimedia Appendix 4 A comparison of the following survey methods for urinary catheter use: manual parsing versus nursing notes.

Multimedia Appendix 5 Results of implementation actions to increase patients’ app use.

Multimedia Appendix 6 Baseline characteristics of the point prevalence surveys.

## Abbreviations

CAUTI: catheter-associated urinary tract infection
EMR: electronic medical record
HAI: health care–associated infection
NeLL: National eHealth Living Lab
PPS: point prevalence surveys
