# Supplementary material for: Reducing Inappropriate Urinary Catheter Use by Involving Patients Through the Participatient App: Before-and-After Study
Source: JMIR Form Res. 2022 Apr 4;6(4):e28983. doi: 10.2196/28983 (PMC9016499; doi:10.2196/28983)
Supplement: Multimedia Appendix 1 [file formative_v6i4e28983_app1.pdf]

This is a Multimedia Appendix to “Reducing Inappropriate Urinary Catheter Use by Involving Patients Through the Participatient App: Before-and-After Study” published in the JMIR Formative Research. For full copyright and citation information see https://doi.org/10.2196/28983

Table S1. Urinary catheter indications

#	Indication	Comment
1	Acute urinary retention or bladder outlet obstruction (≥150 mL)	e.g. neurogenic bladder dysfunction or outlet obstruction by prostate hypertrophy.
2	Accurate measurements of urinary output in patients who are critically ill when required for treatment	In patients with hemodynamic instability such as shock or SIRS, or acute diabetic complications.
3	Before or after surgery according to local protocol	Up to 48 hours**
4	Volume measurements of urine output for diagnostics (24 h urine), which cannot be assessed by other collection strategies	Patient is limited by mental retardation, cognitive capacities or physically handicapped.
5	Assist in healing of open sacral or perineal wounds in patients with urinary incontinence	Both criteria must be met for appropriate indication.
6	Continuous bladder irrigation for haematuria	Bleeding or clots
7	Palliative care for patients who are terminally ill if needed	
8	Other appropriate reason	e.g. surgery on urinary tract and need for wound healing.
9	No appropriate reason	Patient requires immobilization*<br>More than 48 hours post-surgery**
0	Unknown or unclear reason	

Urinary catheter indication scoring of appropriate use according to national and international guidelines [8,9]. * inappropriate unless there is no other feasible method for urinary collection and prolonged immobilization; ** If over 48 hours after surgery, and surgery involves urinary tract, or surgeon indicates the need for a urinary catheter, then scored as ‘other appropriate reason’.
