# Supplementary material for: Reducing Inappropriate Urinary Catheter Use by Involving Patients Through the Participatient App: Before-and-After Study
Source: JMIR Form Res. 2022 Apr 4;6(4):e28983. doi: 10.2196/28983 (PMC9016499; doi:10.2196/28983)
Supplement: Multimedia Appendix 2 [file formative_v6i4e28983_app2.pdf]

This is a Multimedia Appendix to "Reducing Inappropriate Urinary Catheter Use by Involving Patients Through the Participatient App: Before-and-After Study" published in the JMIR Formative Research. For full copyright and citation information see https://doi.org/10.2196/28983

Table S4. Detailed comparison of three survey methods for urinary catheter use, before (T0) and after (T1) implementation of the Participatient app.

	T0 (n=96)	T1 (n=86)	Odds ratio (95% CI)	*P* value***
UC prevalence (A): manual parsing<br>Survey round I<br>Survey round II<br>Survey round III<br>Total	 15 / 33 (45%)<br>10 / 32 (31%)<br>11 / 31 (36%)<br>36 / 96 (37.5%)	 9 / 31 (29%)<br>8 / 30 (27%)<br>6 / 25 (24%)<br>23 / 86 (26.7%)	0.608 (0.324 – 1.144)	0.122
UC prevalence (B): checkboxes<br>Survey round I<br>Survey round II<br>Survey round III<br>Total	 13 / 33 (39%)<br>9 / 32 (28%)<br>13 / 31 (42%)<br>37 / 96 (39%)	 11 / 31 (35%)<br>11 / 30 (37%)<br>7 / 25 (28%)<br>29 / 86 (34%)	0.811 (0.442 – 1.489)	0.499
UC prevalence (C): nurses' notes<br>Survey round I<br>Survey round II<br>Survey round III<br>Total	 9 / 33 (27%)<br>10 / 32 (31%)<br>9 / 31 (29%)<br>28 / 96 (29%)	 9 / 31 (29%)<br>4 / 30 (27%)<br>5 / 25 (20%)<br>18 / 86 (21%)	0.643 (0.325 – 1.270)	0.202
UC Days: ****<br>cumulative<br>median (IQR)<br>mean (standard deviation)	 249<br>2 (1 – 9.75)<br>6.92 (9.213)	 54<br>2 (0.5 – 3)<br>2.35 (2.854)		0.095
No appropriate indication at catheter insertion	10 / 36 (28%)	0 / 23 (0%)	n.a.**	0.006*
No appropriate indication at catheter survey	20 / 36 (56%)	4 / 23 (17%)	0.168 (0.048 – 0.595)	0.004*

Detailed comparison of urinary catheter (UC) use prevalence survey methods: (A) manual parsing of the text in the electronic medical records (EMR); (B) survey of catheter checkbox in the EMR; (C) survey of nurses' pen-and-paper notes. Odds ratios with 95% confidence intervals (95% CI).
* Statistically significant difference. ** Not applicable due to proportion of 0%. *** Associations between categorical variables were tested with Pearson's Chi-square test, and for continuous variables (days) the Mann-Whitney-U test. **** From insertion date to survey date.
