# Supplementary material for: Reducing Inappropriate Urinary Catheter Use by Involving Patients Through the Participatient App: Before-and-After Study
Source: JMIR Form Res. 2022 Apr 4;6(4):e28983. doi: 10.2196/28983 (PMC9016499; doi:10.2196/28983)
Supplement: Multimedia Appendix 3 [file formative_v6i4e28983_app3.pdf]

This is a Multimedia Appendix to “Reducing Inappropriate Urinary Catheter Use by Involving Patients Through the Participatient App: Before-and-After Study” published in the JMIR Formative Research. For full copyright and citation information see https://doi.org/10.2196/28983

Table S5. Comparison of survey methods for urinary catheter use: Manual parsing versus Checkboxes.

Survey methods		Checkboxes		Total			
		no UC	UC				Checkboxes
Manual parsing (gold standard)	no UC	114	9	123		Sensitivity	96.6%
	UC	2	57	59		Specificity	92.7%
Total		116	66	182			

UC = urinary catheter use at time of survey.
