# Supplementary material for: Reducing Inappropriate Urinary Catheter Use by Involving Patients Through the Participatient App: Before-and-After Study
Source: JMIR Form Res. 2022 Apr 4;6(4):e28983. doi: 10.2196/28983 (PMC9016499; doi:10.2196/28983)
Supplement: Multimedia Appendix 4 [file formative_v6i4e28983_app4.pdf]

This is a Multimedia Appendix to “Reducing Inappropriate Urinary Catheter Use by Involving Patients Through the Participatient App: Before-and-After Study” published in the JMIR Formative Research. For full copyright and citation information see https://doi.org/10.2196/28983

Table S6. Comparison of survey methods for urinary catheter use: Manual parsing versus Nursing notes.

Survey methods		Nursing notes		Total			
		no UC	UC				Nursing notes
Manual parsing (gold standard)	no UC	115	8	123		Sensitivity	64.4%
	UC	21	38	59		Specificity	93.5%
Total		136	46	182			

UC = urinary catheter use at time of survey.
