# Supplementary material for: Reducing Inappropriate Urinary Catheter Use by Involving Patients Through the Participatient App: Before-and-After Study
Source: JMIR Form Res. 2022 Apr 4;6(4):e28983. doi: 10.2196/28983 (PMC9016499; doi:10.2196/28983)
Supplement: Multimedia Appendix 5 [file formative_v6i4e28983_app5.pdf]

This is a Multimedia Appendix to “Reducing Inappropriate Urinary Catheter Use by Involving Patients Through the Participatient App: Before-and-After Study” published in the JMIR Formative Research. For full copyright and citation information see https://doi.org/10.2196/28983

Table S2. Results of actions to increase patients’ app use

Week	New Users (n)		Users (n)		Sessions		Avg. Session Duration	Action
**1**	4	10%	5	6%	12	8%	04:05,0	A.
**2**	2	5%	5	6%	8	5%	02:29,0	
**3**	3	7%	5	6%	6	4%	03:52,0	
**4**	3	7%	7	8%	14	9%	03:05,0	
**5**	2	5%	4	5%	10	6%	07:56,0	B.
**6**	2	5%	4	5%	11	7%	01:35,0	
**7**	5	12%	8	9%	11	7%	08:42,0	C.
**8**	3	7%	5	6%	8	5%	04:27,0	
**9**	1	2%	3	4%	3	2%	02:04,0	D.
**10**	2	5%	5	6%	6	4%	00:55,0	
**11**	1	2%	4	5%	9	6%	00:59,0	E.
**12**	2	5%	6	7%	10	6%	01:23,0	E.
**13**	2	5%	4	5%	7	4%	01:01,0	E.
**14**	1	2%	5	6%	14	9%	02:48,0	
**15**	1	2%	3	4%	7	4%	00:47,0	
**16**	1	2%	3	4%	7	4%	00:58,0	F.
**17**	4	10%	6	7%	9	6%	02:06,0	
**18**	3	7%	3	4%	4	3%	06:27,0	
**TOTAL**	**42**		**85**		**156**		**03:13,0**	
**MED (IQR)**	2 (1.3–3)		5 (4–5)		8.5 (7–10.8)		02:17,5	
**AVG (SD)**	2.3 (1.2)		4.7 (1.4)		8.7 (3.1)		03:05,5	

The efficacy of the implementation actions of the app was registered as the number of new users, users and sessions. A ‘new user’ was measured as a new installation on a unique mobile device. A ‘user’ of the app was the use per 24 hours, and each ‘session’ was counted for every instance of use. A = Kick-off with clinical lesson, B = Infographic posters, C* = Recruiting the intake-nurse, D* = Stimulant reminder for the nursing team, E = Support rounds, F = Feedback to nursing team on app use and catheter use prevalence. * Actions C and D were added to the scheduled actions after interim analyses. MED (IQR) = median and interquartile range per week; AVG (SD) = average and standard deviation per week.
