# Supplementary material for: Reducing Inappropriate Urinary Catheter Use by Involving Patients Through the Participatient App: Before-and-After Study
Source: JMIR Form Res. 2022 Apr 4;6(4):e28983. doi: 10.2196/28983 (PMC9016499; doi:10.2196/28983)
Supplement: Multimedia Appendix 6 [file formative_v6i4e28983_app6.pdf]

This is a Multimedia Appendix to "Reducing Inappropriate Urinary Catheter Use by Involving Patients Through the Participatient App: Before-and-After Study" published in the JMIR Formative Research. For full copyright and citation information see https://doi.org/10.2196/28983

Table S3. Baseline characteristics of the point-prevalence surveys

	Total (n=182)	T0 (N=96)	T1 (N=86)	*p*
Sex				.169
Male, n (%)	107 (59%)	61 (64%)	46 (53%)	
Female, n (%)	75 (41%)	35 (36%)	40 (47%)	
Age, years (mean, SD)	63.0 (14.9)	63.9 (14.7)	62.5 (15.3)	

Baseline characteristics of the point-prevalence surveys before (T0), and after the app was introduced (T1).
